# Silicosis-related pleural effusion diagnosed using elemental analysis of the pleural fluid cell block: A case report

**DOI:** 10.1016/j.rmcr.2022.101665

**Published:** 2022-05-11

**Authors:** Shouichi Okamoto, Isao Kobayashi, Hiroshi Moriyama, Mayuka Tanimura, Kotaro Kadoya, Hiroki Ienaga, Toshiaki Kikuchi, Kazuhisa Takahashi

**Affiliations:** a Division of Respiratory Medicine, Juntendo University Faculty of Medicine and Graduate School of Medicine, 2-1-1 Hongo, Bunkyo-ku, Tokyo, 113-8421, Japan; b Department of Respiratory Medicine, Koshigaya Municipal Hospital, 10-32, Higashikoshigaya, Koshigaya, Saitama, 343-8577, Japan; c Department of Respiratory Medicine, Saiseikai Ibaraki Hospital, 2-1-45, Mitsukeyama, Ibaraki, Osaka, 567-0035, Japan; d Department of Respiratory Medicine, NishiNiigata Chuo Hospital, 1-14-1, Masago, Nishi-ku, Niigata, Niigata, 950-2085, Japan; e Department of Respiratory Medicine and Infectious Diseases, Niigata University Graduate School of Medical and Dental Sciences, 757-1 Asahimachi-dori, Chuo-ku, Niigata, 951-8520, Japan

**Keywords:** Cell block, Elemental analysis, Pleural effusion, Silica, Silicosis, CT, computed tomography

## Abstract

Pleural disease in silicosis remains an underrecognized entity. Herein, we describe the case of an 85-year-old man with a 20-year history of silica exposure between the ages of 9–28 years. He presented with bilateral exudative pleural effusions, and chest computed tomography revealed typical silicosis findings. Thoracentesis was performed thrice, but did not reveal the cause of effusion. However, pleural fluid cell-block elemental analysis revealed a silicon compound, suggesting that silicosis-related pleural effusion had developed after a long latency period. Therefore, elemental analysis of the pleural fluid cell block may help diagnose occupational lung diseases with pleural effusion.

## 1 Introduction

Silicosis is one of the most important occupational lung diseases, resulting in irreversible lung fibrosis due to silica inhalation. Silica exposure has also been associated with several comorbidities, including tuberculosis, chronic obstructive pulmonary disease, lung cancer, and autoimmune diseases [1]. Chronic silicosis, the most common type of this disease, has a long latency period of approximately 10–30 years, and some patients remain asymptomatic for as long as 45 years following exposure [2]. Therefore, conducting medical interviews to determine past dust exposure and understanding the radiological findings play important roles in its diagnosis and secondary prevention.

Chronic silicosis typically manifests as multiple small nodules, progressive massive fibrosis, and peripherally calcified mediastinal and hilar lymph nodes on chest computed tomography (CT) [3]. Occupational pleural diseases, characterized by pleural plaques, diffuse pleural thickening, and pleural effusion, are common in patients exposed to asbestos [4]. However, pleural involvement in silicosis is not well recognized. Only four case reports have described pulmonary silicosis with pleural effusion [[5], [6], [7], [8]], and the causal relationship between pulmonary silicosis and pleural effusion remains unclear.

Herein, we describe the first case of chronic silicosis presenting with pleural effusion in which the presence of a silicon compound was proven using elemental analysis of a pleural fluid cell block.

## 2 Case presentation

An 85-year-old man visited our the department of respiratory medicine with left-sided pleural effusion in August 2018. He reported no dyspnea, cough, sputum, chest pain, or fatigue. He was an extensive smoker (75 pack-years) and used a wheelchair. He had a history of coronary artery disease, chronic kidney disease, surgically treated meningioma, and Alzheimer's disease. He regularly visited the department of cardiology at our hospital, but had no family history of cardiopulmonary diseases. He also had a 20-year history of silica exposure between the ages of 9–28 years. He had been a quarry worker for 17 years and a tunnel worker for 3 years subsequently; however, he had never used a dust mask during that period.

On physical examination, he was afebrile, and his oxygen saturation on room air was 96%. Auscultation of the chest revealed normal breath sounds without adventitious sounds. The remainder of the examination findings were unremarkable. Laboratory tests, including a complete blood count, liver function test, thyroid hormone, tumor marker, anti-nuclear antibody, and serum free light chain assays, yielded results within normal limits. QuantiFERON-TB® (an interferon-γ release assay; Qiagen, Hilden, Germany) and assays for autoantibodies associated with connective tissue diseases were negative. Brain natriuretic peptide (77.7 pg/mL) and creatinine (1.67 mg/dL) levels were elevated, but these were almost identical to the levels measured a year ago. Echocardiography revealed a normal ejection fraction, known akinesis of the anteroseptal wall, and no new or worsening wall motion abnormalities.

**Fig. 1:**
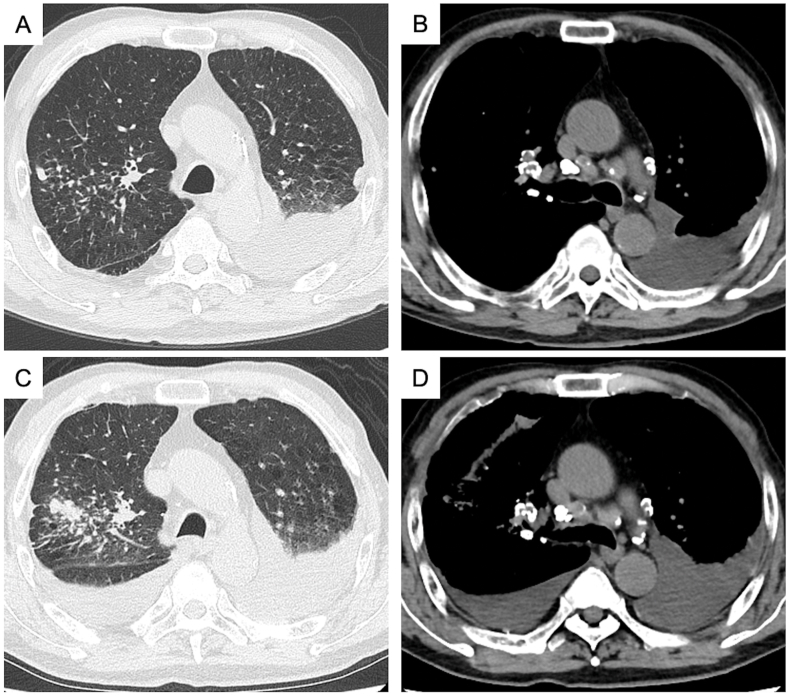
(A–B) and (C–D) are chest computed tomography (CT) images acquired at the first visit and 2 years and 4 months after the first visit, respectively. CT images acquired at the first visit show small nodules in the right upper lung fields (A) and left-sided pleural effusion and egg-shell calcification of the hilar and mediastinal lymph nodes (B). CT images acquired at the second visit show an increase in the sizes of the small nodules (C) and the amount of pleural effusion bilaterally (D).

Chest CT revealed left-sided pleural effusion, small nodules in the right upper lung fields (Fig. 1A), and egg-shell calcification of the hilar and mediastinal lymph nodes (Fig. 1B). CT did not show any characteristic findings of asbestos exposure, such as pleural thickening or plaques. Thoracentesis revealed a clear, yellow-colored, lymphocyte-predominant exudative pleural effusion (fluid protein, 4.3 g/dL; serum protein, 6.6 g/dL; fluid lactate dehydrogenase, 341 U/L; serum lactate dehydrogenase, 230 U/L) (Table 1); cytological analysis revealed that it was a class II effusion (with atypical cells, but no evidence of malignancy), and the Gram staining result was negative (Table 1). The patient received no treatment because the pleural effusion was asymptomatic.

**Table 1:** Pleural fluid analysis.

	1st visit	2nd visit (2 years later)	3rd visit (2 years and 1 month later)
Side	Left	Left	Right
Protein (g/dL)	4.3	4.4	3.4
LDH (IU/L)	341	361	323
Glucose (mg/dL)	100	123	98
ADA (IU/L)	37.8	24.7	39.7
Total cells (/μL)	2900	2600	NA
Neutrophil (%)	9.5	35.5	NA
Lymphocyte (%)	79.5	39	NA
Eosinophil (%)	5.5	19.5	NA
Basophil (%)	0	0	NA
Monocyte (%)	5.5	6	NA
Culture	Negative	Negative	NA

ADA: adenosine deaminase, LDH: lactate dehydrogenase, NA: not applicable.

In December 2020, however, he presented to the emergency department with worsening dyspnea. Bilateral pleural effusion appeared, and the small nodules increased in size (Fig. 1C and D). Left-sided pneumothorax occurred 10 days later. A left-sided exudative effusion was confirmed again following analysis of pleural fluid obtained during needle aspiration for the pneumothorax (Table 1). In January 2021, additional thoracentesis of the pleural effusion on the right side was performed; it revealed a profile that was almost identical to that of the pleural effusion on the left side (Table 1). Thoracoscopy and transbronchial lung biopsy could not be attempted because the patient had a high risk of complications associated with these procedures.

**Fig. 2:**
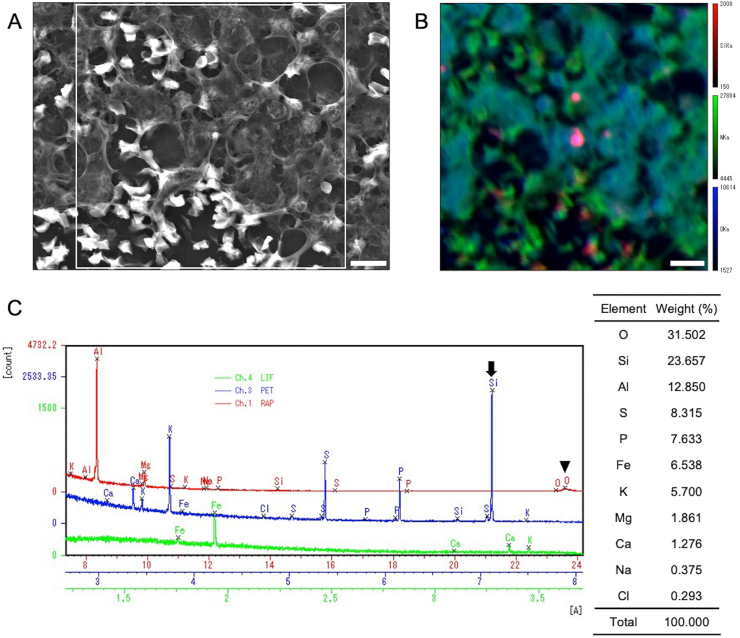
Electron microscopic photograph and elemental analysis using an electron probe microanalyzer equipped with wavelength dispersive spectrometer (EPMA-WDS) of the pleural fluid cell block specimen. (A) A scanning electron microscopy (SEM) image of the cell block specimen. Scale bar: 10 μm. (B) Qualitative colored EPMA-WDS image of an elemental map corresponding to the area indicated by the white rectangle within (A). Silicon, amino nitrogen, and oxygen are colored red, green, and blue, respectively. The distribution of amino nitrogen corresponds to the SEM image (A). The color overlap (red and blue) indicates the coexistence of silicon and oxygen. Scale bar: 10 μm. (C) On the left side of Fig. 2C, one-dimensional analysis of the spot where red and blue overlap, indicated by (B), yields peaks of elements on the curves detected using spectrometer crystal: lithium fluoride (green), penthaerythritol (PET, blue), and rubidium acid phthalate (RAP, red). The peaks of silicon (arrow) and oxygen (arrowhead) are detected on the curves using PET and RAP, respectively. The semiquantitative analysis on the right side of Fig. 2C shows high peaks of oxygen and silicon. Al: aluminum, Ca: calcium, Cl: chloride, Fe: iron, K: potassium, Mg: magnesium, Na: sodium, O: oxygen, P: phosphorus, S: sulfur, Si: silicon, LIF: lithium fluoride; PET: penthaerythritol; RAP: rubidium acid phthalate. (For interpretation of the references to color in this figure legend, the reader is referred to the Web version of this article.)

To clarify the specific etiology of the pleural effusion, a cell block was prepared from 600 mL of the left-sided pleural fluid. The cell block specimen was observed by scanning electron microscopy (Fig. 2A), and elemental analysis using an electron probe microanalyzer equipped with wavelength dispersive spectrometer (JXA-8600, Nihondenshi Co., Ltd., Tokyo, Japan) revealed the coexistence of silicon and oxygen (Fig. 2B). A semiquantitative analysis showed high peaks of oxygen and silicon, suggestive of a silicon compound, potentially silicon dioxide (Fig. 2C).

The elemental analysis thus confirmed the diagnosis of pleural effusion derived from chronic silicosis. The pleural effusion stabilized after treatment with diuretics, and thereafter, the patient received home medical care because of the progression of dementia.

## 3 Discussion

In patients with chronic silicosis, chest CT usually reveals multiple pulmonary nodules, progressive massive fibrosis, hilar and mediastinal lymphadenopathy, calcification of lymph nodes, bullae, and emphysema [3]. However, pleural disease in silicosis has not yet been investigated, especially when compared to that in asbestos exposure. In the present case, we conducted elemental analysis of pleural fluid cell block and ruled out other causes of pleural effusion as much as possible to conclude that chronic silicosis itself can cause pleural effusion.

In a previous study of 110 autopsy cases of chronic silicosis, pleural effusion was detected in 12 (11%) patients with no possible or probable causes, such as pneumonia, cardiac failure, hypoalbuminemia, pneumothorax, and abdominal malignancy, on radiography and CT [9]. However, in that study, pleural fluid analysis, elemental analysis, and examination of birefringent particles compatible with silicosis using polarized light were not performed. Only three case studies have reported pleural fluid analysis results in patients presenting with pleural effusion possibly due to chronic silicosis [5,7,8]. The pleural effusion in all these cases was exudative, and like in our case, it was lymphocyte-predominant in two other cases [5, 7]. In the second thoracentesis of our case, pneumothorax may have considerably affected the fraction of pleural effusion, showing the elevation of the percentages of neutrophils and eosinophils [10]. The presence of silica in the pleural effusion was not analyzed in all reported cases. Interestingly, over 25–50 years had passed from the start of silica exposure to the onset of the pleural effusion in all the three cases [5,7,8]. Our case is unique in that the patient developed pleural effusion with the longest recorded latency period of more than 70 years after silica exposure.

Song et al. reported that exposure to nanoparticles ∼30 nm in diameter for 5–13 months subsequently induced exudative pleural effusions in seven patients working in the same printing plant, and nanoparticles were observed in the patients’ lung tissues, bronchoalveolar lavage fluid, and pleural effusion by transmission electron microscopy [11]. Another report revealed the presence of silica nanoparticles varying in diameter from ∼2 nm to ∼20 nm in the pleural effusions of the same patients by using energy dispersive X-ray analysis [12]. Furthermore, when polyacrylate/silica nanoparticles were intratracheally administered in rats, pleural and pericardial effusions occurred on days 3–5 after the administration, and pleural effusion increased proportionally to the concentration of polyacrylate/silica nanoparticles [13]. These reports and our case support the fact that silica nanoparticles inhaled through the respiratory tract can migrate into the thoracic cavity, regardless of the acute or chronic course of exposure.

The sizes of the silica nanoparticles in the pleural effusions were smaller than those in the lung tissues of the patients reported by Song et al. [12]. Furthermore, silica nanoparticles have been observed in various lung structures, especially in the microlymphatic vessels, as well as in the cytoplasm, nuclei, and organelles of macrophages [12], which are able to migrate into the thoracic cavity in response to specific stimuli [14]. Their report indicated a size-dependent translocation of nanoparticles from the lung to the thoracic lymph nodes, possibly mediated by macrophages. In chronic silicosis, multiple silicotic nodules can be observed protruding from the surface of the visceral pleura [15], and the silicon content is higher in the pleura of patients with silicosis than in that of patients with no clinical evidence of lung disease [16]. In our case, silica might have migrated into the thoracic cavity from the pleura before the presentation of pleural effusion; alternatively, the disruption of the visceral pleura due to pneumothorax might have triggered the migration of silica in the visceral pleura.

Three mechanisms have been speculated as the causes of pleural effusion due to silica nanoparticles: increased pulmonary interstitial fluid levels and permeability of pleural capillaries due to inflammation and the production of reactive oxygen species, mechanical lymphatic obstruction, and damage to the microlymphatic system and its function of fluid reabsorption [13]. Some studies have also shown an association between silica exposure and autoimmune diseases [17,18]. Therefore, silica exposure may act as an immune adjuvant that increases antibody production and causes chronic inflammation. We speculate that our patient developed pleural effusion due to the adjuvant effect of silica; given the long latency since the last silica exposure, it is unlikely that mechanical lymphatic obstruction or lymphatic impairment caused by the silica exposure led to his pleural effusion. However, the patient tested positive for neither anti-nuclear antibodies nor other autoantibodies, and he showed no suggestive findings of connective tissue disease. Therefore, further investigation is warranted to explore the mechanism underlying pleural effusion in chronic silicosis.

Cell block analysis of pleural effusion is widely utilized in cases of malignant pleural disease presenting with effusion, and it is considered a safer alternative to pleural biopsy, especially if the patient is ineligible for biopsy or surgery. However, the diagnostic utility of pleural fluid cell block has rarely been reported in nonmalignant pleural disease, and has been reported in only a few case reports of lymphangioleiomyomatosis [19] and pleural amyloidosis [20]. To the best of our knowledge, this is the first case report of a silicon compound identified via elemental analysis of the pleural effusion.

This study had some limitations. The concentration of silicon in the pleural effusion in our case was not analyzed and compared to those in other diseases, such as malignant pleural effusion, bacterial pleurisy, or benign asbestos pleural effusion. Moreover, we could not estimate the extent of migration of silica from the lungs to the pleural cavity of the patient with silicosis or compare it to that in normal lungs. Furthermore, the pleural fluid was not observed using transmission electron microscopy. Therefore, the presence of nanoparticles in our patient's pleural fluid was not evaluated. However, it is highly unlikely that silicon compounds can be found in the pleural fluid cell block samples obtained from healthy controls without dust exposure.

## 4 Conclusion

We reported a case of chronic silicosis-related pleural effusion that occurred with the longest known latency period after silica exposure. Elemental analysis of a pleural fluid cell block might be a helpful tool in establishing the diagnosis of occupational lung diseases with pleural effusion.

## 5 Declarations of competing interest

The authors declare that they have no competing interests.

## Funding

None.

## Authors’ contributions

SO: study design, collecting data, data analysis, and manuscript writing. IK, MT, KK: collecting data and data analysis. HM: performing elemental analysis and manuscript editing. TK: performing elemental analysis. HI, KT: manuscript writing, reviewing, and editing. All the authors have read and approved the final manuscript.
